# Giant Genital Mass: Unveiling a Unique Case of Folliculocystic and Collagen Hamartoma

**DOI:** 10.7759/cureus.62036

**Published:** 2024-06-09

**Authors:** Hitaishi Mehta, Smriti Gupta, Anuradha Bishnoi, Debajyoti Chatterjee, Keshavamurthy Vinay

**Affiliations:** 1 Dermatology, Post Graduate Institute of Medical Education and Research, Chandigarh, IND; 2 Histopathology, Post Graduate Institute of Medical Education and Research, Chandigarh, IND

**Keywords:** vulval lesion, skin of color, hamartoma, genital mass, folliculocystic and collagenous hamartoma

## Abstract

Folliculocystic and collagen hamartoma (FCCH) is a rare entity, typically documented in males with tuberous sclerosis complex. Here, we report a unique case of FCCH in a 19-year-old female with an unusual presentation in the external genitalia. The patient presented with a progressively enlarging mass over three years, causing difficulties in walking and sitting. Examination revealed a 10 x 15 cm tender, lobulated, skin-colored tumor with comedo-like openings originating from the right labium majus, with satellite lesions on both labia majora. She had no other symptoms or history suggestive of tuberous sclerosis. Histopathological examination showed dilated hair follicles with keratin, perifollicular fibrosis, and thick dermal collagen bands extending into subcutaneous tissue, confirming FCCH. This case underscores the importance of considering FCCH in the differential diagnosis of genital masses, even without classical clinical associations. Our findings contribute to the limited literature on FCCH and highlight the need for further exploration and awareness within the medical community.

## Introduction

Folliculocystic and collagen hamartoma (FCCH) is a rare and relatively recently identified entity, predominantly affecting males with tuberous sclerosis complex. Tuberous sclerosis complex is an autosomal dominant genetic disorder arising from heterozygous mutations in the TSC1 or TSC2 tumor suppressor genes and is marked by the formation of hamartomas in various organs such as the brain, heart, skin, eyes, kidneys, lungs, and liver. Proposed as an atypical variant of collagen hamartoma, FCCH demonstrates unique histopathological and clinical features [1]. The disease typically presents as an exophytic plaque with an elastotic consistency and comedo-like openings, with over half of the cases appearing in infancy or early childhood [2]. The head is the most common site of involvement, with genital involvement never previously reported. Histopathologically, this entity is characterized by dermal collagen deposition, dilation of the infundibular portion of the hair follicles, and concentric fibrosis around the hair follicles [3]. To date, nearly 20 cases of FCCH have been documented in the literature, with approximately 90% associated with tuberous sclerosis and a small minority occurring independently of it [1,4]. In this report, we present a unique case of FCCH in a female, highlighting an unusual site of occurrence and clinical profile.

## Case presentation

**Figure 1:**
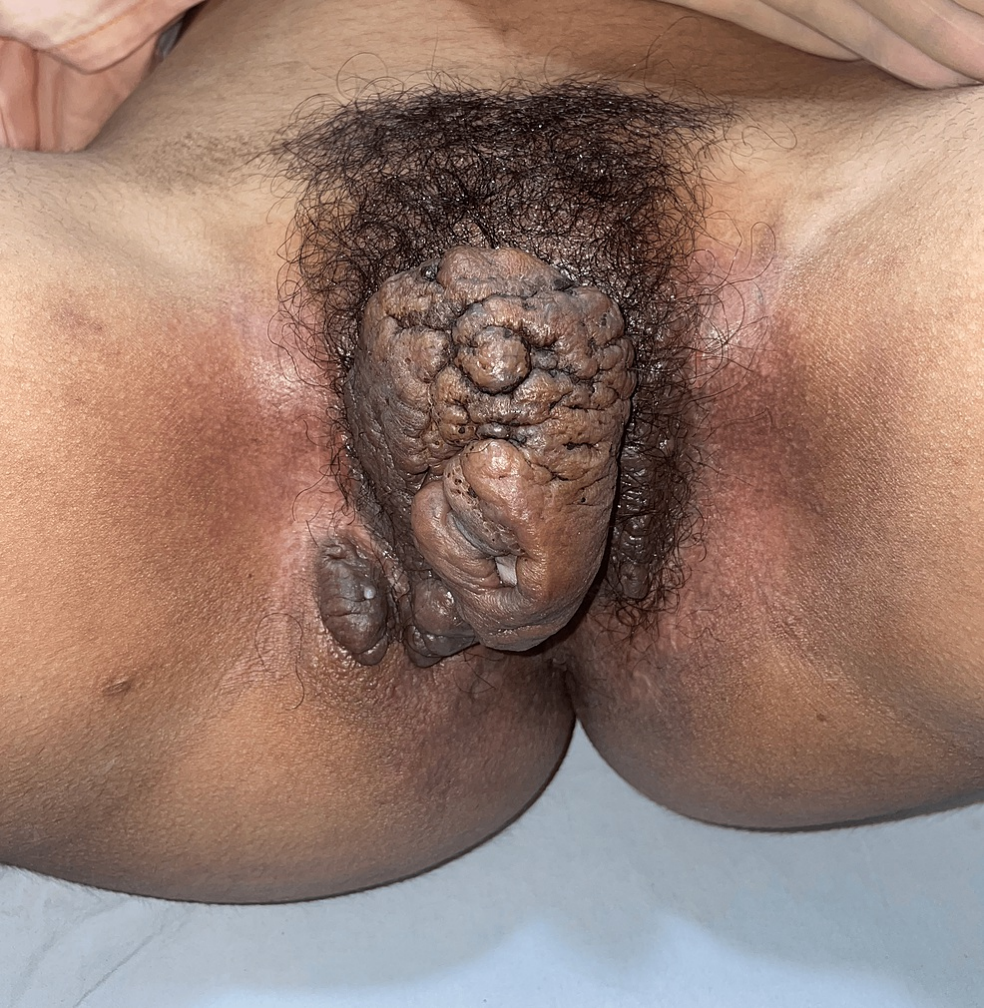
Illustration of a lobulated, non-tender, skin-colored, and firm genital mass with comedo-like openings, originating primarily from the right labium majus and obstructing the external genitalia

A 19-year-old, otherwise healthy girl sought medical attention due to a progressively growing mass involving the external genitalia, causing difficulties in walking and sitting over the past three years. The lesion had started as a nodule five years ago over the right vulval lip and slowly progressed to involve the entire genitalia. Physical examination revealed a tender, soft-to-firm, lobulated, pedunculated, and skin-colored tumor, with a rubbery consistency and comedo-like openings. Measuring approximately 10 x 15 cm, the mass originated primarily from the right labium majus, obstructing the external genitalia (Figure 1).

**Figure 2:**
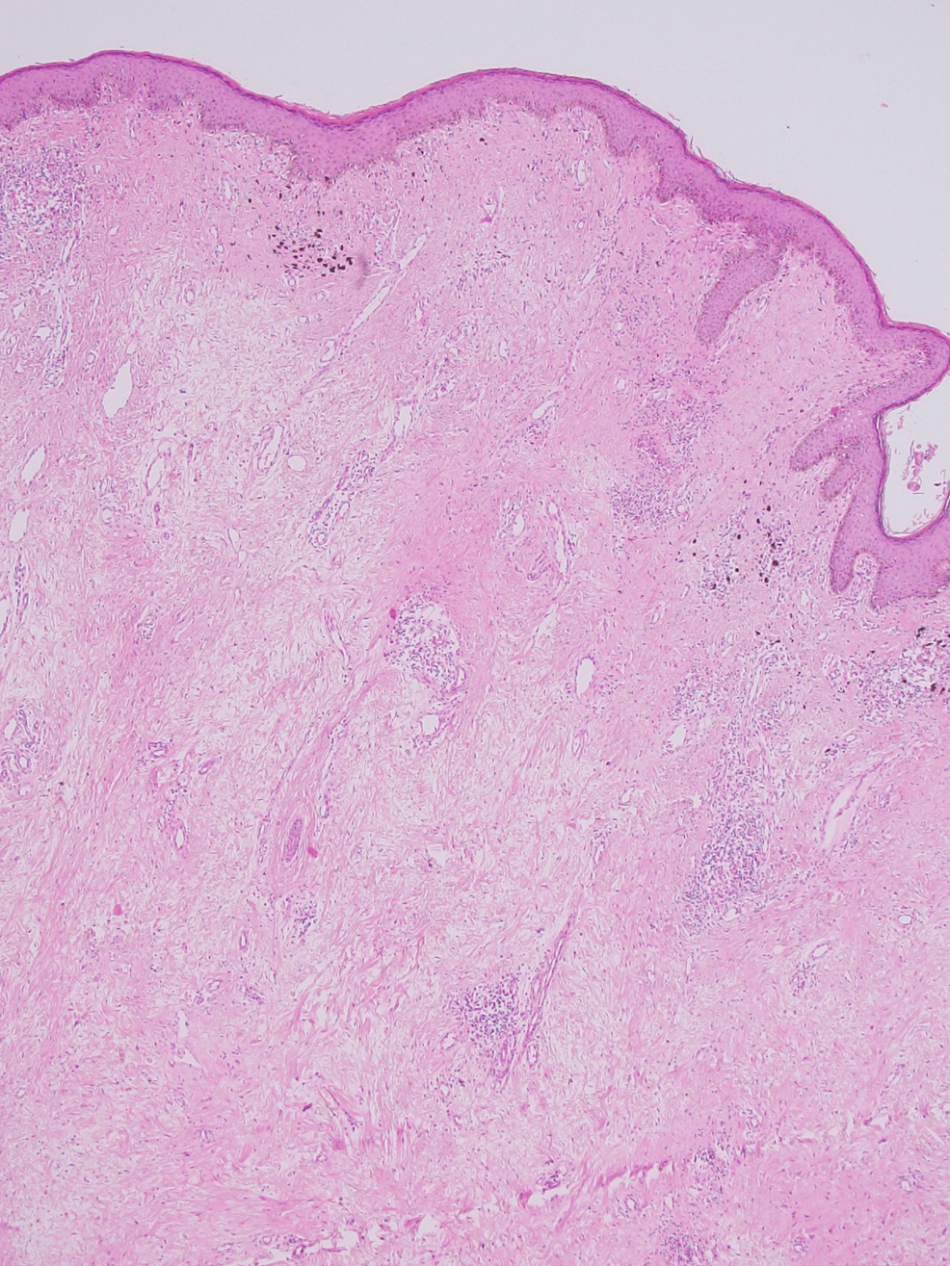
Scanning photomicrograph showing epidermal acanthosis and hyperkeratosis, with dermal infiltration of collagen fibers and dilated hair follicles (Hematoxylin & Eosin, 10x)

Satellite lesions were observed on the same labia as well as on the left labium majus. The patient reported no other cutaneous or systemic complaints. The patient history revealed no seizures, developmental delays, intellectual disabilities, or autism spectrum disorder. On cutaneous examination, there were no hypomelanotic macules, facial angiofibromas, shagreen patches, or similar symptoms in family members. She was not sexually active, denied gastrointestinal complaints, and exhibited no signs or symptoms indicative of tuberous sclerosis. There was no lymphadenopathy or pedal edema; ultrasound pelvis showed no abnormality. Considering the location and the size of the growth, a comprehensive differential diagnosis, including hidradenitis suppurativa, condyloma acuminata, connective tissue nevi, cutaneous Crohn’s disease, folliculosebaceous cystic hamartoma (FSCH), and elephantiasis nostras verrucosa was considered. Low-power magnification revealed the epidermis with irregular acanthosis and hyperkeratosis (Figure 2).

**Figure 3:**
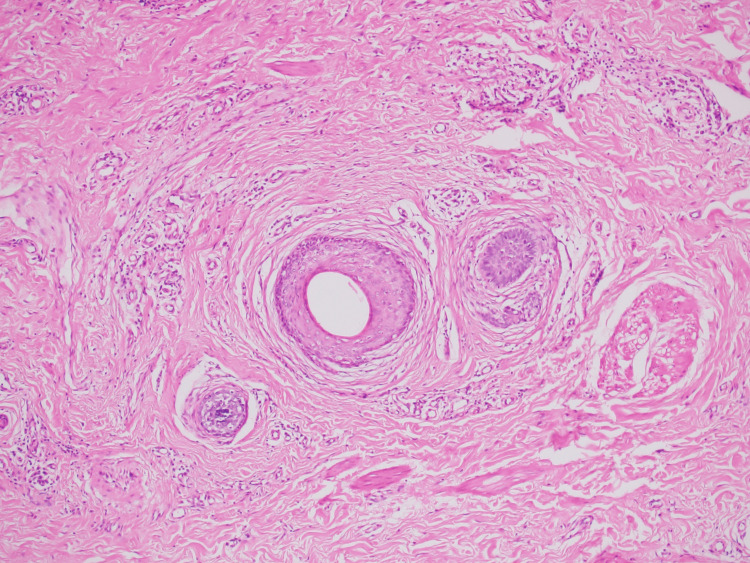
Photomicrograph of hair follicles surrounded by concentric fibrosis (Hematoxylin & Eosin, 10x)

Additionally, variably sized dilated hair follicles containing keratin were observed (Figure 3).

**Figure 4:**
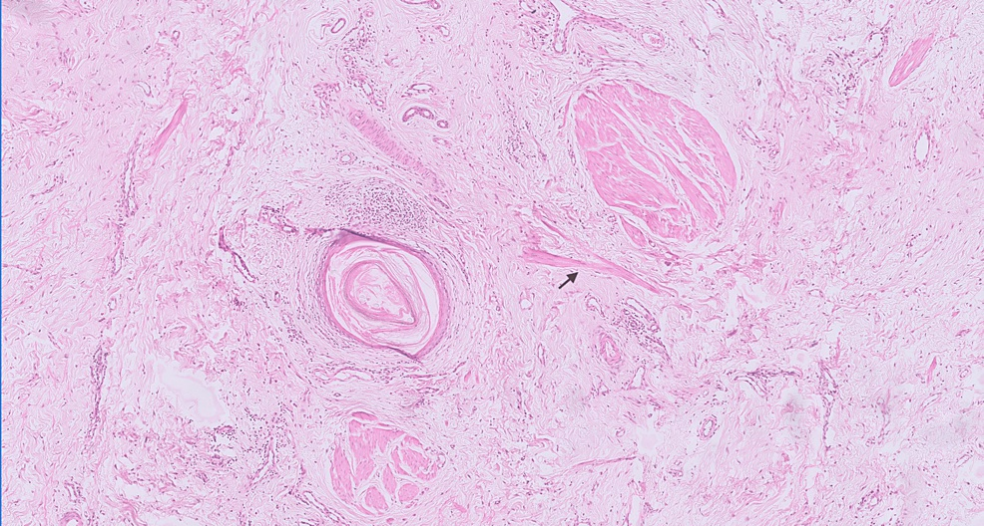
Photomicrograph depicting perifollicular fibrosis and thick dermal collagen bands arranged haphazardly in the dermis, with dilated blood vessels extending into the subcutaneous tissue. A few unpaired arrector pili are also observed, indicated by black arrows (Hematoxylin & Eosin, 40x)

Collagen fibers were arranged haphazardly, along with a few unpaired arrector pili muscles (Figure 4).

**Figure 5:**
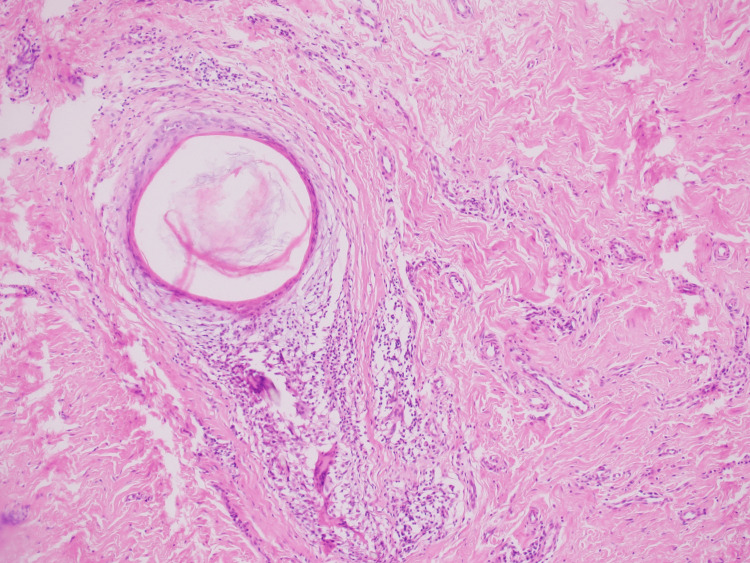
Histopathological examination showing variably sized dilated hair follicles containing keratin, surrounded by collagen fibers (Hematoxylin & Eosin, 40x)

There was accompanying perifollicular fibrosis and thick dermal collagen bands extending into the subcutaneous tissue (Figure 5).

**Figure 6:**
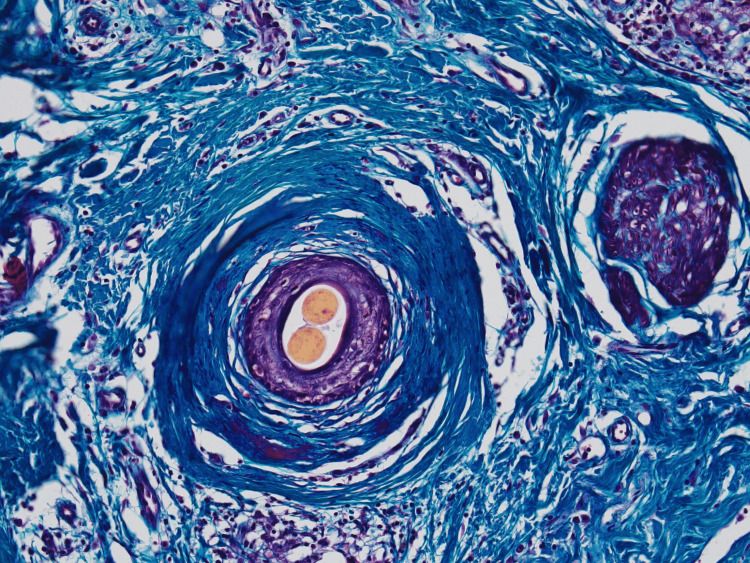
Masson’s trichrome stain highlighting increased collagen fibers arranged concentrically around the hair follicle and in a haphazard pattern within the surrounding dermis (magnification 60x)

Masson's trichrome stain highlighted the concentrically arranged collagen fibers surrounding hair follicles (Figure 6).

This led to the establishment of a diagnosis of FCCH. The patient is scheduled for surgical debulking.

## Discussion

FCCH is an exceedingly rare hamartoma that has received limited attention in the medical literature, primarily documented in patients with tuberous sclerosis and displaying a male predilection. The prevailing theory suggests the involvement of TSC1/TSC2 genes and sex hormones in its pathogenesis, yet the emergence of cases in females and those without tuberous sclerosis contradicts this hypothesis [5]. A case series studying patients with FCCH in tuberous sclerosis patients identified heterozygous pathogenic variants in the TSC2 gene in tumor tissue samples, without evidence of a TSC2 second hit, suggesting a potential genotype-phenotype correlation [2].

These lesions typically manifest at birth or early infancy, affecting areas such as the scalp, face, abdomen, or thighs. Clinically, they present as painless plaques or exophytic masses with irregular surfaces, adorned with comedonal openings, variable cysts, and occasional extrusion of keratinous or purulent material [6]. The distinctive histopathological features of FCCH encompass collagen deposition in the form of bundles spanning the dermis and reaching the subcutaneous tissue, without an increase in ground substance. Additional characteristics include concentric perifollicular and peri-eccrine fibrosis as well as cysts lined by infundibular epithelium and filled with keratin. Notably, the occurrence of FCCH in the genital region, though unprecedented in the literature, exhibited characteristic morphology and histopathology in our case. Genetic testing could not be performed in this case due to economic constraints. However, we strongly believe that tuberous sclerosis complex is unlikely in this case, given the absence of symptoms, signs of tuberous sclerosis, and a lack of family history of the condition.

The other differential diagnosis was collagenoma, a hamartomatous proliferation of collagen, histologically exhibiting variable elastin fiber content. While there are commonalities such as perifollicular fibrosis, a notable distinction lies in the absence of infundibular cyst formation in collagenoma, a feature distinctively observed in FCCH [1]. Considering alternative hamartomatous proliferations, FSCH was also contemplated, particularly as it has been documented in the vulvar region, although it commonly appears on the face as well-defined papules or nodules [7]. Histopathologically, FSCH differentiates from FCCH by featuring dilated infundibular structures filled with keratin, sebaceous secretion, and rudimentary hair shafts. These infundibular channels may exhibit radiating sebaceous ducts and subsequent glandular structures. Characterized by a mixed stroma of fibroplasia, mucin, nerve fibers, fibrocytes, smooth muscle fibers, and blood capillaries, FSCH primarily confines itself to the dermis without extending involvement to the overlying epithelium and underlying subcutis [7].

Vulvar symptoms can represent rare extraintestinal manifestations of Crohn’s disease, often presenting as exophytic or bulbous growths. These manifestations typically involve lymphatic growth and may be associated with non-caseating granulomas [8]. Given the absence of systemic symptoms and histopathological findings inconsistent with Crohn’s disease, this diagnosis was ruled out. Warts were excluded due to the absence of sexual activity, a non-keratinized surface, and the microscopic absence of koilocytes. The absence of hyperkeratosis, mossy texture, and the presence of only a few satellite lesions amid normal skin effectively ruled out the possibility of elephantiasis nostras verrucosa, a conclusion supported by histopathological findings [9]. Additionally, the absence of comedones in other regions, the lack of sinus tracts and scarring, along with the non-involvement of other flexures, diminished the likelihood of hidradenitis suppurativa.

Management of FCCH primarily involves surgical excision, particularly when the lesion causes discomfort due to mass effect or keratinaceous discharge. Malignant transformation has not been reported to date, and the potential for recurrence remains uncertain due to a lack of long-term studies.

## Conclusions

In conclusion, our case highlights a unique occurrence of FCCH in a female, presenting at an atypical site without any association with tuberous sclerosis. This case underscores the importance of including FCCH in the differential diagnosis of genital masses, even when traditional clinical associations are absent. The rarity of FCCH, particularly in females and at unusual sites, calls for heightened clinical awareness and consideration in similar cases. Our report contributes to the limited literature on FCCH, emphasizing the need for further research to better understand its pathogenesis and clinical spectrum. This expanded knowledge can facilitate more accurate diagnoses by providing clinicians with a broader understanding of its manifestations and improving management strategies by informing evidence-based treatment options.
